# Is Sex an Underrated Risk for Relapse in Substance Use Disorders?

**DOI:** 10.3390/brainsci16010071

**Published:** 2026-01-03

**Authors:** Annette Bohn, Niels Graf, Norbert Scherbaum, Daniel Deimel, Henrike Schecke

**Affiliations:** 1 LVR-University Hospital, Department of Psychiatry and Psychotherapy, Medical Faculty of the University of Duisburg-Essen, 45147 Essen, Germany; 2 German Institute of Addiction and Prevention Research, Catholic University of Applied Sciences Nordrhein-Westfalen, 50668 Koeln, Germany; 3 Faculty of Social Science, Nuremberg Institute of Technology Georg Simon Ohm, 90489 Nuremberg, Germany

**Keywords:** sexualized substance use, substance use disorders, sexuality, stimulants, relapse

## Abstract

**Background:** Sexualized substance use (SSU) describes the use of psychotropic substances in the context of sexual activity. Less is known about the role of sexualized substance use among individuals with substance use disorders (SUD) and its effect on the course of the disorder, e.g., regarding relapses after abstinence. **Methods:** A convenience sample of individuals undergoing SUD rehabilitation in Germany was surveyed. A questionnaire asked about SSU, sex as a risk factor for relapse, and the importance of sexuality in treatment. **Results:** N = 490 (30.1% female) participated; 55% of men and 63% of women reported SSU, and 56.5% of heterosexual and 82.9% of homosexual men reported SSU (*p* < 0.017; r = 0.20). Stimulant users are more likely to report SSU than alcohol (*p* < 0.001) and sedative users (*p* < 0.001; r = 0.296 and r = 0.261). Furthermore, 15% of women and 18% of men consider sexual activity a risk factor for relapse; homosexual men (65%) consider it significantly more often than heterosexual men (14%), while 41.2% of heterosexual women and 55% of homosexual women consider it a factor. Additionally, 27.4% of heterosexual and 69.4% identified sexuality as an important topic for therapy, while 19.8% of heterosexual women, 30% of homosexual women, 13.5% of heterosexual men, and 47.2% of homosexual men reported that sexuality had been addressed in their therapy. **Conclusions:** SSU was reported by individuals with a SUD who were undergoing rehabilitation treatment. Furthermore, patients consider sexual activity as a potential risk factor for relapse, with this being particularly the case for stimulant users. The topic of sexuality is highly important for patients and should, therefore, be given greater consideration in therapy in the future.

## 1. Introduction

Sexualized substance use (SSU) describes the use of psychotropic substances in the context of sexual activity. As a research topic, the interplay between substance use and sexuality has so far been limited to specific target groups like the “chemsex” of men, who have sex with men [1], sexual risk behavior of adolescents and young adults [2], or certain aspects of sexual behavior like compulsive sexual behavior [3] or sexual dysfunction [4]. For a non-clinical sample, the “*Global Drug Survey*” [5] investigates adults of different ages, gender identities, and sexual orientations regarding substance use patterns. Here, 31.2% of men and 22.9% of women reported any SSU. Alcohol, cannabis, and MDMA were the most commonly used substances in a sexual context. More homosexual men reported SSU than bisexual men, who were more likely to report SSU than heterosexual men. More bisexual women reported SSU compared to homosexual women, who did not show a difference from heterosexual women. Different substances were also analyzed regarding their specific impact on sexual experience, which covered both enhancing and disinhibiting aspects. While a general limitation of the Global Drug Survey is that it attracts mainly participants who are prone to substance use, these findings still show that SSU seems to be a relevant topic.

Considerably less is known about the role of sexualized substance use among adult individuals with substance-related disorders and their effects on the course of the disorder, for example, with regard to relapses after achieving substance abstinence. There are only two studies investigating the relationship between different substances and their influence on sexual experience in populations of people in treatment for SUD [6,7]. In the study by Boosma-Bleeker and Blauw, 60% of all patients reported enhancing effects of substances on their sexual experience, while 60% also reported a reduction in a sexual domain under the influence of substances. Almost a quarter of the participants indicated that their sexual thoughts, feelings, or behaviors were often influenced by their substance use, and 10% reported difficulties with separating their substance use from their sexual behavior. Ten percent also experienced substance cravings after having sexual thoughts and feelings. The main findings of both studies show similar results regarding the perceived effects on sexuality between different substance groups. Both studies compared groups of patients who mainly consumed opiates or sedatives, alcohol, or stimulants. Those who used stimulants reported the highest impact on their sexual experience, while those who consumed alcohol reported a stronger influence compared to those who consumed opiates or sedatives.

Regarding the motivations for SDU, the studies by Boosma-Bleeker and Blauw, Rawson et al. and Lawn et al. offer insights into sexual domains that are affected by substance use. More often, there is a focus on positive and enhancing effects, but there are also findings for using substances to reduce adverse experiences. A study comparing motives to drink alcohol or use drugs in a sexual context identified that the main motives for drinking alcohol were to facilitate sexual encounters, while the main motives for using drugs were improvement in sexual experiences and performance [8]. A recent study from Canada found four motivations for SSU via exploratory factor analysis: *satisfaction and sensations*, *increasing sexual self-esteem*, *mitigating distress*, and *increasing sexual response and function* [9]. Another Canadian study found that higher substance use severity scores were associated with a higher presence of sexual motivations for substance use [10]. Jones and Lorenz found that SSU was not more common for women who are unpartnered, but is just as likely for women in relationships [11]. Thus far, there is no standardized instrument for assessing sexual motivations.

In substance use disorders, relapse is a frequent occurrence [12]. Relapse rates occurring a year after treatment for a SUD vary from 40 to 80% of patients [13,14]. Various influencing factors for a relapse have been studied: e.g., the type of substance [15], length of treatment [16,17], and different sociodemographic factors [18,19]. There is also research on interpersonal and ecological factors affecting relapse [20,21]. There are different theoretical models that explain how substance relapses evolve. The cognitive behavioral model from Marlatt [22] states that in a high-risk situation, a person’s coping response determines the occurrence of a relapse. High-risk situations, according to the model, include interpersonal situations, of which sexual encounters could be a subcategory. Hence, in relapse prevention for people with SUDs, sexual situations could be an important aspect to cover, especially for those who have previously used substances in sexual settings. Thus, the inclusion of sexuality as a topic in treatment programs for SUDs seems to be a necessary and important addition. Thus far, there is only one qualitative study from Norway that explores sexual situations as a potential risk for relapse, and in this case, it is in a specific population of people who were incarcerated or recently released from prison [23]. The author concluded from the interviews that the prospect of having sex without the influence of substances was a relevant and potentially distressing situation for the interviewees, which could result in a relapse and should, therefore, be considered when working on relapse prevention strategies. In light of the evidence that substance use and sexuality are linked in various ways, and that sexual situations could potentially pose a risk for relapse, it seems crucial to address sexuality as a topic in treatment programs for SUD. However, the few existing studies indicate the opposite—sexuality, as a topic, is hardly broached in SUD treatment [24,25].

### Study Aims

This present study examines a sample of SUD patients undergoing rehabilitation treatment. The aim was to find out whether patients considered sexual activity to be a potential relapse situation. It also asks whether sexuality should be addressed as a topic in therapy and whether this has already been done during their rehabilitation treatment. It was hypothesized that the patients subjectively view sexuality as a potential relapse situation and that the topic of sexuality has rarely been addressed in their current rehabilitation.

## 2. Materials and Methods

### 2.1. Sampling

In Germany, a high proportion of rehabilitative therapy for SUDs takes place in inpatient treatment in specialized rehabilitation clinics. Outpatient rehabilitation is linked to the condition of a stable social environment and accounts for a significantly lower proportion of rehabilitation cases. Inpatient rehabilitation treatment usually lasts 8 to 12 weeks for alcohol dependence and 12 to 24 weeks for illegal substance dependence. Outpatient rehabilitation lasts 12 months. The costs are generally covered by the pension insurance providers or, in some cases, by health insurance. The diagnostic criteria according to ICD-10 are mandatory in the German healthcare system. The main diagnosis for admission to rehabilitation treatment must be a substance dependency, as defined by the ICD; secondary diagnoses may also include harmful use, as defined by the ICD-10.

Data for the convenience sample were collected from June 2020 to the end of January 2021 from ten different rehabilitation clinics for substance use disorders. The majority of clinics focused on various illegal substances and alcohol. Each clinic specializes in the treatment of people with cannabis and cocaine-related disorders, respectively. One clinic offered a special treatment track for chemsex users (mostly MSMs). One clinic only accepted men for treatment. Participation in the study was voluntary after informed consent was given. As an incentive, the participants each received a voucher for an online store in the amount of EUR 15. The survey was conducted using a questionnaire in paper-and-pencil format.

### 2.2. Instruments/Measures

The questionnaire was designed by the study group, which consisted of social and health scientists and psychologists. The questionnaire was developed based on expert consensus but was not psychometrically validated. The first part of the questionnaire covered sociodemographic data and information on gender identity, sexual orientation, and substance use, as well as the duration and setting of rehabilitation. The second part included questions on substance use in a sexual context and an instrument on the participant’s motives for substance use in a sexual setting, for which a German translation of an instrument from the study by Bosma-Bleeker and Blaauw (2018) [7] was used. Questions were also asked about the frequency of various sexual activities and the number of sexual partners had. In addition, standardized questionnaires on sexual satisfaction, sexuality-related fears, and sexual self-confidence were included. However, these were not included in the analysis for this article. There were four key questions for this analysis. *“Did you use your main substance explicitly in a sexual context?”* was answered using a five-point Likert scale (*never, nearly never, sometimes, often, very often*). The question *“Do you think that sexual activity could pose a risk of relapse?”* was answered using a five-point Likert scale (*no, rather no, partly, rather yes, yes*). The question *“Would it be important for you to talk about sexuality as part of your current rehabilitation treatment?”* was also answered using a five-point scale (*not important, somewhat important, partly important, very important*). The question *“Has sexuality been discussed as part of your current rehabilitation?”* was answered using a three-point scale (*no, somewhat, yes*).

Participants were recruited by the treatment team at the clinic where the patients underwent rehabilitation. The questionnaire could be completed independently by the participants in a paper-and-pencil version. Participants were given a stamped envelope to enable them to return the completed questionnaires anonymously via mail.

### 2.3. Statistical Analysis

Most analyses differentiated by binary gender and sexual orientation. For further analysis, groups were formed according to the substance primarily used. A distinction was made between people who were in rehabilitation exclusively for an alcohol-related disorder and people who primarily used other substances and as well as alcohol. A differentiation was made between sedative and stimulant substances. The group of sedative substances included cannabis, benzodiazepines, opioids (both heroin and opioid analgesics), and GHB/GBL. The group of stimulants included cocaine, amphetamines, methamphetamines, and cathinone. The polydrug use group included people who consumed alcohol as well as sedatives, as well as stimulant substances.

IBM SPSS 27 was used for statistical analysis; for nominal data, the Chi^2^ test and Mann–Whitney U test were used. A *p*-value that was smaller than 0.05 was taken as statistically significant, and the effect sizes (Pearson’s r) were categorized according to Cohen. Effects for multiple group testing were reduced by using the Bonferroni correction method.

## 3. Results

### 3.1. Sample

In total, N = 490 participated in the survey. The mean age was 35.7; one-third (30.1%) were female. Regarding sexual orientation, 87.2% stated that they were heterosexual, and 12.9% stated that they were homosexual or bisexual. Further sample characteristics and information on the primary substances used are displayed in Table 1 and Table 2. Due to the paper-and-pencil nature of the survey, which does not allow for a forced-choice response format, the total figures may vary (e.g., for net income).

### 3.2. Substance Use in Sexual Settings

A total of 55% of men and 63% of women stated that they use their primary substance for sexual activities. Differentiated by sexual orientation, 56.5% of heterosexual men and 82.9% of homosexual men used substances explicitly for sexual activity. There was a significant difference between both groups; homosexual men were significantly more likely (*p* < 017; r = 0.20) to use substances for sexual activities. Respectively, 71.3% of heterosexual and 65% of homosexual women reported using substances for sexual activities. Those groups did not differ significantly.

The following results were obtained with regard to the different main substance groups. A Dunn–Bonferroni corrected ANOVA was conducted to control for multiple testing (Table 3). These show that the groups of primary stimulant users and polyvalent users differ significantly (*p* < 0.001) from alcohol users and primary sedative users. Primary stimulant users are more likely to report consuming substances specifically for sex at least as frequently as alcohol users (*p* = 0.000) and sedative users (*p* = 0.000), with effect sizes of r = 0.296 and r = 0.261, respectively, which represent medium effects. Mixed effects were shown for primary polyvalent users compared to alcohol users (*p* = 0.001; r = 0.259) and sedative users (*p* = 0.008; r = 0.221), representing medium and small effects, respectively. All gropu comparisons are displayed in Table 4.

### 3.3. Sexual Activity as Relapse Risk

Among the women participants, 15% stated that sexual activity was a potential risk situation for a relapse; among the male respondents, 18% stated this. There was no significant difference between male and female participants. Differentiated by sexual orientation, among men, 14% of heterosexual and 65% of homosexual men stated that sexual activity could be a risk for relapse. There was a significant difference between the groups; homosexual men reported significantly more often that sexual activity could be a risk factor for substance use relapse. Amongst the women, 15% of heterosexual women and 25% of the homosexual women stated that sexual activity could be a risk for relapse. There was no statistically significant difference for women.

### 3.4. Importance of Addressing Sexuality in Therapy

Among the women participants, 41.2% of heterosexual and 55% of homosexual women stated that addressing sexuality in the current therapy would be important/very important to them. For the men, 27.4% of heterosexual and 69.4% of homosexual men identified sexuality as an important/very important topic for their therapy. Differences based on sexual orientation only resulted in a significant difference for men. In contrast to the subjective importance of sexuality as a topic in therapy, 19.8% of heterosexual women, 30% of homosexual women, 13.5% of heterosexual men, and 47.2% of homosexual men reported that sexuality has been addressed in their current therapy. Sexuality was significantly more addressed in therapy with homosexual men (*p* = 0.004; Cramer’s V = 0.251). All results are displayed in Table 5.

## 4. Discussion

The “SUBSEX” project gathered data on the sexualized substance use of patients in substance use disorder rehabilitation in Germany. Although these data were collected under the conditions of the COVID-19 pandemic, 490 people were interviewed.

More than half of the respondents stated that they had also used their main substance specifically for sexual activity. Homosexual men and heterosexual women stated this most frequently.

These results show that sexualized substance use in individuals with a substance use disorder is a phenomenon that has been insufficiently addressed in the research literature so far. Focusing solely on MSMs in the context of chemsex or adolescents is, therefore, falling short. Individuals with a SUD should also be included in research and therapeutic care for sexualized substance use.

Sexual activity was identified as a perceived relapse risk situation for both women and men. The SUBSEX study results suggest that sexual situations could be high-risk situations for relapse for some individuals with a SUD. According to Marlatt’s relapse model, relapse in sexual activity occurs when an interpersonal risk situation is combined with inadequate coping mechanisms on the part of the individual. This applies, in particular, to stimulant users and sexual minorities, especially MSMs.

Various factors that generally promote substance use are discussed for MSMs and other sexual minorities. Meyer’s minority stress model [26] states that specific stressors come into play for sexual minorities, which require psychological and social resources. The discrepancy between one’s own sexual orientation and heteronormative society, and the repeated experience of discrimination, act as specific stressors that require additional individual coping strategies. Substance use can be interpreted as a dysfunctional coping strategy. If substance use is ceased or reduced, alternative strategies are needed to cope with minority stress. Building supportive social networks or individual stress management strategies could be helpful starting points.

A subgroup of male participants who were surveyed had also sought treatment due to a chemsex-associated SUD. Chemsex consumption patterns highlight the close association between sexual activity and substance use. Substance use is sometimes an integral part of sexual activity [27]. Additionally, there is an overlap with the group of stimulant users, as stimulants are increasingly consumed in the chemsex context [28]. Social norms that favor substance use in subgroups of the MSM community could also play a role in the particular link between sexuality and substance use [29]. In addition, the use of dating apps, which are more frequently used by chemsex users [29], may, through their algorithms, encourage encounters between people who are also open to substance use in a sexual context. People who wish to abstain would therefore have to “retrain” their dating app algorithms in order to see fewer risky user profiles.

It was found that users of primarily stimulant substances, in particular, identify sexual activity as a risk situation. This corresponds with previous study results, which also found a closer association with sexual activity among users of stimulants [7]. This may also be associated with the effects of the substance itself, as stimulants such as amphetamines, cocaine, and methamphetamines can have a direct effect on sexual desire and (risk) behavior in men [30] and women [31]. In the case of relapse, the expectation of a more intense sexual experience could also play a role here, which might challenge abstinence. In the treatment of stimulant users, the topic of sexuality as a risk factor for relapse should, therefore, be addressed as a priority.

Developing effective coping strategies for maintaining substance abstinence is one of the most important components of any cognitive behavioral therapy for a SUD. About half of the female participants stated that addressing their sexuality as an issue in therapy would be important or very important to them. Just over a quarter of heterosexual men and two-thirds of homosexual men found it important or very important to discuss their sexuality in therapy. In contrast to the subjective importance of the topic of sexuality for therapy, the proportion of people who reported that sexuality was addressed in therapy was significantly lower. Among both women and men, sexuality was discussed more often among non-heterosexual individuals, and was particularly frequently discussed among homosexual men. Sexuality was most rarely discussed with heterosexual men in the context of therapy. As such, different reasons why sexuality is not addressed in addiction therapy can be discussed: Patients may hesitate to talk proactively about sexuality unless the therapist asks them directly. There may be uncertainty about whether sexuality is an appropriate topic in treatment when the focus is on substance use disorders. The topic of sexuality may also be perceived as shameful, or there may be a lack of language and forms of expression to talk about sexual content. The latter can be relevant for both patients and therapists and can create barriers. Therapists and other healthcare professionals may also feel uncomfortable discussing sexual issues with patients, as there is little training on sexuality and sexual health in therapeutic education. The unequal gender ratio between therapists and patients could also have an influence. In therapeutic professions in addiction services, young female therapists often primarily encounter predominantly male patients of middle age. This could lead to shame-related barriers on both sides and may have contributed to the fact that sexuality was discussed least with heterosexual men.

Uncertainty and a lack of knowledge could also play a role in the area of sexual diversity. Sexuality and relationships beyond cis-heteronormative and monogamous couples may not be given sufficient consideration. Unconscious biases and prejudices toward sexual diversity on the part of the therapist may also play a role here, undermining a trusting therapeutic alliance and discouraging patients from discussing sexuality. Sexual therapy training courses and training on sexual diversity could also be helpful tools for enabling therapists to engage in dialogue with their patients on sexual topics. Other implementable interventions could include routinely taking a sexual history in SUD therapy and incorporating psychoeducational elements on the interplay between substance use and sexuality into group therapy.

In order to broaden the perspective on sexual diversity, routine questionnaires used in clinical practice could also be adapted, for example, by including different sexual and gender identities or forms of partnership. This would also signal to patients an openness and awareness of issues of sexual diversity.

In addition to the importance of engaging in therapeutic dialogue about sexuality in the first place, research findings on motives for consumption in sexual contexts provide a starting point for discussing the topic of sexual activity as a relapse situation. Deshaies et al. (2024) [9] identified *satisfaction and sensations*, *increasing sexual self-esteem*, *mitigating distress*, and *increasing sexual response and function* as key motives for engaging in sexualized substance use among individuals with a SUD. These can be important therapeutic starting points on the path to sexuality while abstaining from substances. When working therapeutically on critical situations that could lead to a relapse, a distinction can be made between avoidable situations (e.g., visiting a bar) and situations that are difficult to avoid (visiting a German supermarket that also usually sells alcohol). For the latter, it is important that patients in therapy learn strategies to successfully cope with these situations without experiencing a relapse. Since sexuality plays an important role in the lives of many people, sexual activity is also one of the situations for which a functional strategy should be developed during abstinence. Rehabilitation treatment provides a good opportunity for developing these strategies. Findings and strategies from sex therapy can also be useful here.

Limitations: This study reported on sexualized substance use in a sample of patients who were undergoing rehabilitation treatment for substance use disorders and provided different ideas on how to discuss sexuality and acknowledge this as an important topic in the treatment of substance use disorders.

However, the results should be interpreted in light of several limitations. Methodologically, it is important to note that the instrument also included questions that were not psychometrically evaluated but were merely developed based on the expert consensus of the study group. This represents the most significant limitation in interpreting the results. A lack of reliability and validity of the questionnaire cannot be ruled out. This should lead to a very cautious interpretation of the results. Operationalizing the core statements with single questions is also problematic. However, as one of the few studies on sexualized substance use among people with a SUD, this study can serve as a basis for further research. The development of an appropriately psychometrically evaluated instrument should precede further studies in this area.

In addition, the selection of the ten rehabilitation clinics is not representative of all clinics in Germany, which means that selection bias cannot be ruled out. However, an anonymous self-report questionnaire survey somewhat reduced the risk of social desirability bias when it came to the sensitive topics of sexuality and substance use. An imbalance in the gender ratio in the sample may reduce the significance for female users, although having one-third female participants fairly accurately reflects the typical distribution in addiction treatment in Germany. This does not indicate a systematic effect in data collection to the detriment of women.

When evaluating the individual substance classes separately (sedative vs. stimulant), it cannot be ruled out that there may have been overlaps and inaccuracies. This may weaken the assertion that sexuality poses a risk of relapse, especially for stimulant users.

The COVID-19 pandemic, during which the study was conducted, may have had an impact not only on data collection, but it is also possible that the use of rehabilitation services changed during this period, as did sexual activity prior to therapy.

Due to the cross-sectional design of this study, it is only possible to make conclusions about the subjectively perceived individual risk of relapse in connection with sexual activity as experienced by the respondents. In order to find out whether sexual activity actually promotes relapse, longitudinal studies would be necessary.

When interpreting the results relating to homosexual men, it should be noted that generalizability to MSMs with all SUDs is significantly limited. In the study, MSMs are overrepresented at 8.3% compared to the male German general population (2.7% [32]). Both may be due to the fact that one clinic has a special track for MSMs with chemsex use disorders. Hence, the findings on MSMs are most likely limited to a subgroup of MSMs who are seeking treatment for chemsex use disorders and not for all MSMs with an SUD.

It can, therefore, be concluded that sexual orientation is not the decisive factor here. The close functional link between substance use and sexuality may have a greater effect on chemsex users than, for example, MSMs with an alcohol use disorder.

## 5. Conclusions

Sexualized substance use is reported by individuals with a substance use disorder undergoing rehabilitation treatment. A significant proportion of patients consider sexual activity to be a potential risk factor for relapse. This is particularly the case for stimulant users. Subjectively, the topic of sexuality is highly important in addiction therapy and should be given greater consideration in therapy in the future.

## Figures and Tables

**Table 1:** Sample characteristics.

		N	%
Age (M = 35.7, SD 10.7 min = 18, max = 73)	<20	10	2.0
	20–29	152	31
	30–39	161	32.9
	40–49	104	21.2
	50–59	50	10.2
	>60	13	2.7
Gender	female	147	30.1
	male	339	69.5
	other	2	0.4
Sexual orientation and gender (only binary gender included)	heterosexual women	124	25.7
	homo-/bisexual women	22	4.6
	heterosexual men	297	61.5
	homo-/bisexual men	40	8.3
Main substance	Alcohol	116	23.7
	Cannabis	96	19.6
	Amphetamines	78	15.9
	Cocaine	114	23.3
	Opioids	31	6.3
	Chemsex substances	27	5.5
	(methamphetamine, GHB/GBL, mephedrone, ketamine)		
	others	28	5.7
Native language	German	410	86.7
	others	63	13.3
Net income (in EUR)	<1000	195	43.3
	1000–<2000	149	33.3
	2000–<3000	63	14.0
	>3000	42	9.4
Setting rehabilitation	outpatient	125	25.5
	inpatient	365	74.5

**Table 2:** Description of sexualized substance use of 490 patients in substance use disorder rehabilitation treatment.

Did You Use Your Main Substance Explicitly in a Sexual Context?									
	Never/Nearly Never		Sometimes		Often/Very Often				
	N	%	N	%	N	%	Mann–Whitney U Test	Asymptotic Significance	Effect Size Pearson’s r
gender									
female	34	24	19	13	90	63	n = 143	*p* = 0.102	-
							mean rank: 249.40		
male	81	25	65	20	179	55	n = 325		
							mean rank: 227.95		
sexual orientation									
women									
heterosexual	10	10.6	17	18.1	67	71.3	n = 124	*p* = 0.869	
							mean rank: 73.46		
homo-/	2	10	5	25	13	65	n = 22		
bisexual							mean rank: 73.7		
men									
heterosexual	54	21.3	56	22.1	143	56.5	n = 287	*p* = 0.017	0.20
homo-/							mean rank: 158.6		
bisexual	2	5.7	4	11.4	29	82.9	n = 38		
							mean rank: 196		

**Table 3:** Substance use as a risk factor for substance relapse in 490 patients in substance use disorder rehabilitation treatment.

Sexual Activity as a Risk for Substance Relapse									
	No/Rather No		Partly		Rather Yes/Yes				
	N	%	N	%	N	%	Mann–Whitney U Test	Asymptotic Significance *p*	Effect Size Pearson’s r
gender									
female	96	65	29	20	22	15	n = 147	*p* = 0.803	-
							mean rank 239.29		
male	231	69	45	13	59	18	n = 335		
							mean rank 242.47		
sexual orientation									
women									
heterosexual	79	64	27	22	18	15	n = 124	*p* = 0.974	-
							mean rank 73.46		
homo-/	16	73	2	9	4	25	n = 22		
bisexual							mean rank 73.75		
men									
heterosexual	212	72	39	13	42	14	n = 293	*p* < 0.001 *	0.23
							mean rank 160.33		
homo/	18	45	6	15	26	65	n = 40		
bisexual							mean rank: 221.48		

**Table 4:** Pairwise comparison of relapse risk in different main substance groups in 490 patients in substance use disorder rehabilitation treatment. (* and possibly additionally alcohol).

	z	Standard-Error	Bonferroni Corrected *p*	Effect Size Pearson’s r
Group comparisons (Mann–Whitney U Test)				
only alcohol vs. sedating substances *	−9.438	16.945	1.000	-
only alcohol vs. polysubstance use	−68.187	18.616	0.001	0.259
only alcohol vs. stimulating substances *	−77.723	16.365	0.000	0.296
sedating substances * vs. polysubstance use	−58.749	18.321	0.008	0.221
sedating substances * vs. stimulating substances	−68.285	16.028	0.000	0.261
polysubstance use vs. stimulating substances *	9.536	17.786	1.000	-

**Table 5:** Sexuality as a topic during the rehabilitation of 490 patients in substance use disorder rehabilitation treatment.

**Would It Be Important for You to Talk About Sexuality as Part of Your Current Rehabilitation Treatment?**												
	not important		somewhat important		partly		important		very important		Chi^2^	Effect size (Cramer’s V)
	N	%	N	%	N	%	N	%	N	%		
women												
heterosexual	14	14.4	25	25.8	18	18.6	21	21.6	19	19.6	4.525	-
homo-/bisexual	4	20	5	25	-	-	5	25	6	30	*p* = 0.104	
men											25.651	0.298
heterosexual	64	25.4	57	22.6	62	24.6	43	17.1	26	10.3	*p* < 0.001	
homo-/bisexual	2	5.6	4	11.1	5	13.9	7	19.4	18	50.0		
**Has Sexuality Been Discussed as Part of Your Current Rehabilitation Treatment?**												
	no		somewhat		yes							
	N	%	N	%	N	%						
women												
heterosexual	36	37.5	41	42.7	19	19.8					0.0733	-
homo-/bisexual	9	45	5	25	6	30					*p* = 0.693	
men												
heterosexual	137	54.6	80	31.9	34	13.5					11.068	
homo-/bisexual	13	36.1	6	16.7	17	47.2					*p* = 0.004	0.251

## Data Availability

Original data can be requested from the corresponding author. Public access is not possible for ethical reasons, as the consent of the test subjects for public access to the data was not obtained.
